# Pyrroroquinoline Quinone (PQQ) Improves the Quality of Holstein Bull Semen during Cryopreservation

**DOI:** 10.3390/ani14202940

**Published:** 2024-10-11

**Authors:** Hai Wang, Kexiong Liu, Weibin Zeng, Jiahua Bai, Linli Xiao, Yusheng Qin, Yan Liu, Xiaoling Xu

**Affiliations:** 1 Institute of Animal Husbandry and Veterinary Medicine, Beijing Academy of Agriculture and Forestry Sciences, Beijing 100097, China; 18899596501@163.com (H.W.); liukexiong2023@163.com (K.L.); bai_jiahua@126.com (J.B.); xiao1990linli@163.com (L.X.); blackberrysheng@163.com (Y.Q.); 2 College of Animal Science and Technology, Shihezi University, Shihezi 832000, China; zwbdky@126.com

**Keywords:** pyrroroquinoline quinone (PQQ), reactive oxygen species (ROS), oxidative damage, cryopreservation, bull semen

## Abstract

**Simple Summary:**

Cryopreserved semen, widely utilized in the artificial insemination of domestic animals, encounters challenges due to oxidative damage. This study investigated the effects of the antioxidant PQQ on the semen quality of Holstein bulls during cryopreservation and elucidated the associated mechanisms. Our findings revealed that PQQ significantly enhanced sperm motility, membrane integrity, acrosome integrity, and ATP levels, while simultaneously reducing MDA and ROS levels. Additionally, increased PGAM2, CAPZB, CAT, SOD1, and GPX1 protein expressions were also observed in the PQQ-treated group. Based on these results, PQQ not only improved semen quality but also mitigated oxidative stress, thereby enhancing the efficacy of sperm cryopreservation in bulls.

**Abstract:**

Cryopreserved semen is extensively utilized in the artificial insemination (AI) of domestic animals; however, suboptimal conception rates due to oxidative damage following AI continue to pose a challenge. The present study investigated the effects of Pyrroroquinoline Quinone (PQQ), a novel antioxidant, on the semen quality of Holstein bulls during cryopreservation, as well as its potential molecular mechanisms. Semen samples were diluted with varying concentrations of PQQ (0, 50 μmol/L, 100 μmol/L, 150 μmol/L) prior to cryopreservation. Following the freeze–thaw process, a comprehensive evaluation was conducted to assess sperm motility, plasma membrane integrity, acrosome integrity, and the levels of reactive oxygen species (ROS), malondialdehyde (MDA), and adenosine triphosphate (ATP). Western blot analysis was employed to examine the levels of proteins including PGAM2, CAPZB, CAT, SOD1, and GPX1. Notably, the inclusion of 100 μmol/L PQQ significantly enhanced sperm motility, membrane integrity, and acrosome integrity post freeze–thawing (*p* < 0.05). Furthermore, the group treated with 100 μmol/L PQQ exhibited reduced levels of MDA and ROS (*p* < 0.05), while ATP levels were significantly elevated (*p* < 0.05). Interestingly, treatment with 100 μmol/L PQQ resulted in decreased consumption of PGAM2, CAPZB, CAT, SOD1, and GPX1 proteins in sperm after freeze–thawing, compared to the control group (*p* < 0.05). These findings indicate that PQQ treatment enhances the quality of bull semen, mitigates oxidative stress damage, and ultimately improves the efficacy of sperm cryopreservation.

## 1. Introduction

As an advanced modern technology, artificial insemination (AI) has largely supplanted traditional natural mating methods for domestic animals. The practice of sperm cryopreservation allows for the long-term preservation of gametes while maintaining satisfactory quality, viability, and developmental potential, thus enhancing the expansion of AI reproductive techniques. In the context of breeding and selection programs, the cryopreservation of sperm in domestic animals is of paramount importance, as it accelerates genetic improvement through the implementation of AI procedures [1]. This method is particularly advantageous when inseminating selected or multiple females with semen from a male exhibiting desirable genetic traits [2]. Additionally, the cryopreservation of gametes has been utilized as a strategy to protect species from extinction and to facilitate the reproduction of endangered animals [3].

During the freezing process, various factors can modify the lipid composition of spermatozoa, diminish motility, stimulate the release of enzymes, and ultimately lead to sperm cell death. Specifically, sperm produce excessive concentrations of reactive oxygen species (ROS) due to the unphysiological storage conditions, which induces oxidative stress [4]. Prior research has established that oxidative stress impairs sperm morphological and physiological characteristics, including sperm motility, membrane integrity, acrosome integrity, DNA integrity, and subsequent fertilization potential [5,6]. Furthermore, excessive accumulation of ROS has been shown to have detrimental effects on the structure of sperm mitochondria, as reported by Ashrafi and Schwarz in 2013 [7]. It also results in a reduction in mitochondrial ATP production in sperm, as evidenced by the study conducted by Chianese and Pierantoni in 2021 [8]. Due to their minimal cytoplasmic volume, spermatozoa often exhibit a scarcity of endogenous antioxidants, which are crucial for protecting cellular structures from potential ROS-induced damage [9,10]. Additionally, during sperm processing, alterations in physiological characteristics, particularly those resulting from the dilution or removal of seminal plasma, lead to a significant depletion of endogenous antioxidants, including glutathione peroxidase (GPX), glutathione reductase (GSH) [11], superoxide dismutase (SOD) [12], and catalase (CAT) [13]. This depletion, constituting a primary source of antioxidant protection for spermatozoa [10], heightens their vulnerability to oxidative damage [14].

A variety of antioxidants have been employed to mitigate the harmful effects of oxidative stress and to enhance sperm quality, viability, and post-fertilization developmental potential [6,15]. Pyrroloquinoline quinone (PQQ; 4,5-Dihydro-4,5-dioxo-1H-pyrrolo[2,3-f]quinoline-2,7,9-tricarboxylic acid; molecular formula is C14H6N2O8), a water-soluble vitamin-like factor, with significant high pro-oxidant activity as a redox cofactor [16], has been identified in plant products [17], mammalian organs [18], and bacteria [19,20]. Recent studies have demonstrated that PQQ possesses a range of biological properties, including antioxidant effects, mitochondrial activation, anti-inflammatory capabilities, neuroprotection, and the facilitation of growth, development, and reproduction in animals. In boar sperm, it has been observed that both ATP production and the mitochondrial transcription system are impaired as ROS levels increase. In this context, PQQ serves as a valuable agent for preserving mitochondrial DNA integrity and maintaining the linear motility of sperm [21]. In aging layer breeder roosters, supplementation with 1 mg/kg dietary PQQ.Na_2_ as an antioxidant has been shown to improve sperm quality and enhance antioxidant activity [22]. In rams, during storage at 4 °C, PQQ has been effective in protecting sperm quality by quenching ROS levels, thereby reducing ROS damage and maintaining mitochondrial function, which preserves the sperm’s fertilization capability [23].

Previous research has indicated that PQQ significantly affects semen quality. However, there are a lack of data regarding the impact of PQQ on thawed frozen Holstein bull spermatozoa, and the underlying mechanisms remain poorly understood. Therefore, the current study aims to investigate the effect of PQQ on Holstein bull sperm during cryopreservation and to conduct a preliminary exploration into the relevant mechanisms involved.

## 2. Materials and Methods

### 2.1. Semen Collection and Processing

The Holstein bulls, aged 2 to 3 years and exhibiting excellent physical condition and robust sexual vitality, were procured from the Beijing Dairy Cattle Center (Beijing Capital Agribusiness & Food Group Co., Ltd., Beijing, China). To mitigate potential individual variations, semen from two bulls was combined for subsequent treatment during each repetition, and the entire experiment was conducted in triplicate to ensure consistency and accuracy. All six bulls were utilized for semen collection using the artificial vagina method. Briefly, one bull was positioned as a stationary animal, while the target bull was led to it for a trial. After the target bull mounted, its penis was quickly inserted into the sperm collection cup, and the cup was held in place until ejaculation occurred, after which the cup was slowly raised and the glass tube removed. The bull semen used in this study exhibited a milky white or slightly grayish hue, with a distinct yet non-offensive odor. Fresh sperm total motility exceeded the 85% threshold, while the rate of sperm morphological deformity remained below 10%. The semen underwent a dilution process (1:1 *v*/*v*) using the EMCARE™ Holding Solution (ICPbio Reproduction, Glenfield, New Zealand) and was promptly transported to the laboratory within a two-hour timeframe, maintaining a temperature of 17 °C. During transportation, the semen was gently agitated every thirty minutes to reduce the risk of sperm aggregation.

### 2.2. Cryopreservation and Thawing of Semen

Upon arrival at the laboratory, the semen was tested for sperm density using the NucleoCounter device, manufactured by Chemometec in Denmark. Following this analysis, the semen was diluted in a 1:5 (*v*/*v*) ratio using Optidyl extender (IMV Technologies, L’Aigle, France), a commercially available extender containing egg yolk. This dilution aimed to achieve a sperm concentration of approximately 1.0–1.5 × 10^8^ sperm per milliliter. Prior to dilution, different concentrations of PQQ (0 μmol/L, 60 μmol/L, 120 μmol/L, and 180 μmol/L) were added to the Optidyl extender. Consequently, the diluted semen was allocated into four distinct groups based on PQQ concentration: 0 μmol/L, 50 μmol/L, 100 μmol/L, and 150 μmol/L.

The processed semen was carefully dispensed into 0.25 mL straws (Minitüb GmbH, Offenbach, Germany) and securely sealed with polyvinyl chloride powder. The straws underwent a cooling process at 4 °C for four hours. Sperm quality assessments were conducted prior to freezing. Following this, the straws were positioned approximately 4 cm above the liquid nitrogen level in nitrogen vapor for 15 min. Subsequently, the straws were immersed in liquid nitrogen at −196 °C. They remained stored in liquid nitrogen until the thawing process was initiated. After one week of freezing, the straws containing the frozen semen were thawed in a water bath at 38 °C for 30 s. The thawed semen was then subjected to further analysis.

### 2.3. Semen Quality Analysis

Thorough assessments of the sperm’s total motility, as well as the integrity of the plasma membrane and acrosome, were conducted on the sperm samples both before freezing and after thawing.

Evaluation of sperm total motility: The total motility of sperm was assessed using the Computer-Assisted Sperm Analysis (CASA) System (Sperm Class Analyzer CASAS-QH-III; Tsinghua Tongfang, Beijing, China). In this procedure, 5 µL of the sperm samples was placed into a pre-warmed chamber (one-time sperm counting slides, Yufan, China). Prior to microscopic examination, the chamber was heated to 37 °C for 30 s. Subsequently, three random visual fields, each containing a minimum of 400 spermatozoa, were selected to record the total motility data for each sample.

Detection of sperm plasma membrane integrity: The assessment of sperm membrane integrity was conducted using a combination of fluorescent probes, specifically propidium iodide (PI) and Hoechst33342, as previously detailed in the literature [24]. In brief, 100 μL of semen was co-incubated with 20 μL of 12 μmol/L PI and 10 μL of a 1 μg/mL solution of Hoechst33342 at 37 °C for 10 min in the dark. Subsequently, the semen was examined using a fluorescence inverted microscope (Olympus CH 30, RF-200, Tokyo, Japan). During the assessment of sperm cell membrane integrity, we observed that sperm with an intact plasma membrane emitted blue fluorescence, while those exhibiting pink fluorescence demonstrated a compromised plasma membrane, indicating that these cells were non-viable. To ensure an accurate evaluation of cell membrane integrity, we assessed the staining properties of at least 200 spermatozoa in each microscopic field.

Detection of sperm acrosome integrity: The integrity of the sperm acrosome was assessed using a double staining technique that employed lectin derived from Arachis hypogaea (peanut agglutinin), conjugated with fluorescein isothiocyanate (FITC-PNA) and PI [25]. Briefly, each semen sample was treated with 100 μg/mL FITC-PNA and 10 μg/mL PI staining at 37 °C for 15 min. The sperm were subjected to two rounds of washing via centrifugation at 328× *g* for 5 min each, then resuspended in DPBS, and subsequently, the semen was examined under a fluorescence inverted microscope (Olympus CH 30, RF-200, Tokyo, Japan). During this process, at least 200 spermatozoa from each sample were evaluated to ensure the accuracy and reliability of our findings.

### 2.4. Measurement of Sperm ROS Levels

To assess sperm ROS levels, an ROS assay kit (S0033S, Beyotime Institute of Biotechnology, Shanghai, China) was utilized according to the manufacturer’s guidelines. In brief, sperm samples, comprised of 1.0 × 10^6^ sperm per group were prepared with three biological replicates and incubated with DCFH-DA (10 μmol/L) at 38 °C in a dark setting for 25 min. After this incubation period, the samples were centrifuged at 1500× *g* for 4 min, and the obtained pellet was washed twice with PBS. Subsequently, the pellet was resuspended in PBS, and the concentration of ROS was measured using a fluorescence spectrophotometer (Tecan Infinite^®^200Pro, Vienna, Austria) with excitation and emission wavelengths set at 488/525 nm. Variations in sperm ROS levels were evaluated by comparing the ratio of fluorescence intensity to sperm density within each group.

### 2.5. Measurement of Sperm BCA Concentrations, ATP, and MDA Level

The evaluation of sperm ATP and malondialdehyde (MDA) concentrations was carried out using the Bicinchoninic Acid (BCA) Protein Concentration Determination Kit (P0012), ATP assay kit (S0026), and MDA assay kit (S0131S), all provided by Beyotime Institute of Biotechnology (Shanghai, China) [26]. Initially, 500 μL of PBS was incorporated into thawed semen, which was subsequently centrifuged at 2000× *g* for 5 min to remove impurities. After this step, 250 µL of RIPA buffer was introduced, and the resulting mixture underwent ultrasonication for lysis at 4 °C for a duration of 5–10 min, followed by centrifugation at 12,000× *g* for 10 min. The supernatant was then extracted for further analysis. In the BCA assay, standard solutions (0 to 0.5 mg/mL in increments of 0.025 mg/mL) were combined with the BCA detection reagent and the test samples. The resulting mixtures were incubated at 37 °C for 30 min. BCA concentrations were subsequently determined using a fluorescence spectrophotometer set at a wavelength of 595 nm (Tecan Infinite^®^200Pro, Vienna, Austria). For the ATP content measurement, the ATP detection solution was mixed with standard solutions (0.01, 0.03, 0.1, 0.3, 1, 3, 10 μM) and the test samples. To eliminate background ATP, the detection well was first treated with the ATP detection solution before adding the test samples for ATP content assessment using a fluorescence spectrophotometer at 595 nm (Tecan Infinite^®^200Pro, Vienna, Austria). In the MDA assay, standard solutions (1, 2, 5, 10, 20, 50 μM) were combined with the MDA detection solution and the test samples. The mixtures were incubated at 100 °C for 15 min and allowed to cool to room temperature, and then MDA levels were quantified using a fluorescence spectrophotometer at 532 nm (Tecan Infinite^®^200Pro, Vienna, Austria).

### 2.6. Western Blotting

The total sperm protein extraction process was conducted in a sodium dodecyl sulfate (SDS) sample buffer. Sperm precipitation was supplemented with 40 µL of RIPA, 10 µL of 10% SDS, 50 µL of 4 × loading buffer, 1 µL of protease inhibitor, and 1 µL of PMSF, and the mixture was thoroughly mixed. The protein concentration was subsequently measured using the previously mentioned BCA method. Semen lysates containing 15 µg of protein were resolved by 12% SDS polyacrylamide gel electrophoresis (SDS-PAGE) and transferred onto PVDF membranes at a constant current of 200 mA for 90 min. The membranes were blocked with a blocking solution from Beyotime (Beyotime Institute of Biotechnology, Shanghai, China) and incubated overnight at 4 °C with primary antibodies as detailed in Table 1. Following the incubation, the membranes were washed three times with TBST. Secondary antibodies conjugated with horseradish peroxidase (HRP) were then incubated with the membranes for 2 h at room temperature, followed by three additional washes with TBST. β-actin served as a loading control. The membranes were processed with Super ECL Plus, and the resulting Western blotting signals were captured using a Bio-Rad gel imaging system (Bio-Rad Laboratories, Hercules, CA, USA). Each protein was detected on individual PVDF membranes, and the intensities of the blotting signals were quantitatively analyzed by outlining the relevant bands on the film using Image Lab software version 6.0.1.

### 2.7. Statistical Analysis

The statistical analyses were conducted rigorously using SPSS software version 26.0 (SPSS, Inc., Chicago, IL, USA). Data were analyzed through one-way analysis of variance, followed by the LSD post hoc test for pairwise comparison. Graphical representations were created using Graph Pad Prism software version 9.0 (GraphPad Software, San Diego, CA, USA). The results are presented in a concise and standardized format as mean ± standard deviation (SD), with a significance level of *p* < 0.05 established to indicate statistically significant difference.

## 3. Results

### 3.1. Effects of PQQ on the Quality of Holstein Bulls Sperm before Freezing

**Figure 1:**
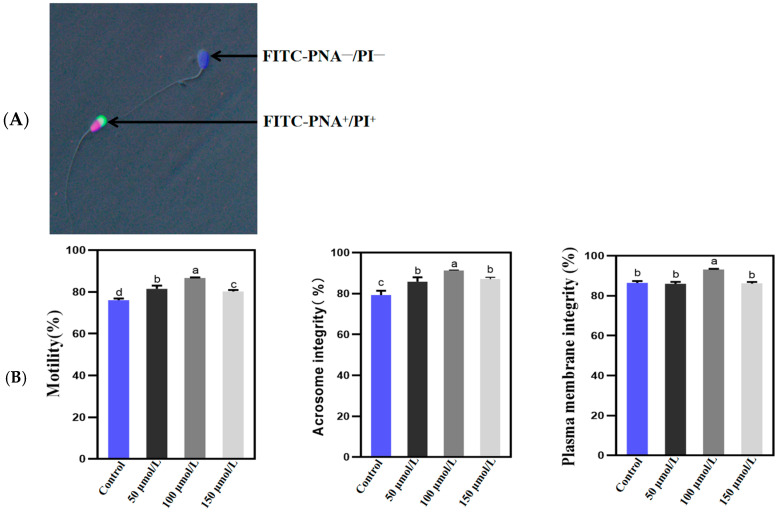
Effects of varying concentrations of PQQ on the quality of Holstein bull sperm prior to freezing. (**A**) Representative images illustrating the acrosome and plasma membrane integrity of bull sperm. (**B**) Statistical analysis of the total motility, acrosome integrity, and membrane integrity of Holstein bull sperm quality before freezing. Control: no PQQ treatment; PQQ: 50, 100, 150 µmol/L PQQ/1.0 × 10^8^ sperm. Values are presented as mean ± SD (*n* = 3); columns with different lowercase letters indicate significant differences (*p* < 0.05).

The comprehensive assessment of Holstein bull sperm prior to the freezing process, which includes total motility, plasma membrane integrity, and acrosome integrity, is illustrated in Figure 1. The results revealed that the application of varying concentrations of PQQ treatment significantly enhanced the sperm total motility and acrosome integrity before freezing (*p* < 0.05). Additionally, treatment with 100 µmol/L PQQ resulted in a significant increase in plasma membrane integrity prior to freezing (*p* < 0.05).

### 3.2. Effects of PQQ on the Quality of Frozen–Thawed Holstein Bulls Sperm

**Figure 2:**
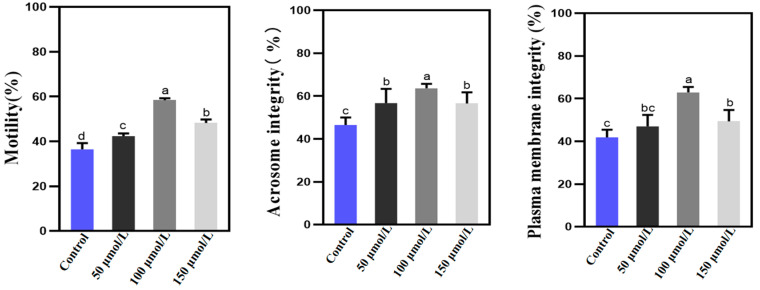
Effects of various concentrations of PQQ on the quality of frozen–thawed Holstein bull sperm. This figure presents the statistics regarding the total motility, acrosome integrity, and plasma membrane integrity of Holstein bull sperm quality following thawing. Control: no PQQ treatment; PQQ: 50, 100, and 150 µmol/L PQQ/1.0 × 10^8^ sperm. Values are expressed as mean ± SD (*n* = 3); columns with different lowercase letters indicate significant differences (*p* < 0.05).

Subsequently, we conducted a comprehensive examination of the post-thaw total motility of Holstein bull sperm, alongside an assessment of acrosome integrity and plasma membrane integrity (Figure 2). Notably, the application of various concentrations of PQQ resulted in a significant enhancement in total motility of post-thaw sperm (*p* < 0.05). Additionally, the proportion of acrosome-intact sperm in the three PQQ treatment groups exceeded that of the control group, demonstrating statistical significance (*p* < 0.05). Furthermore, treatment with 100 µmol/L PQQ led to a marked improvement in the overall plasma membrane integrity of the frozen–thawed sperm, which also achieved statistical significance (*p* < 0.05). These findings highlight the efficacy of PQQ treatment in preserving sperm quality and integrity during the freezing and thawing process. The trends observed regarding the effects of PQQ on the quality of frozen–thawed Holstein bulls sperm were consistent with those of sperm prior to freezing.

### 3.3. Effects of PQQ Treatment on ROS, MDA, and ATP Levels of Frozen–Thawed Holstein Bull Sperm

**Figure 3:**
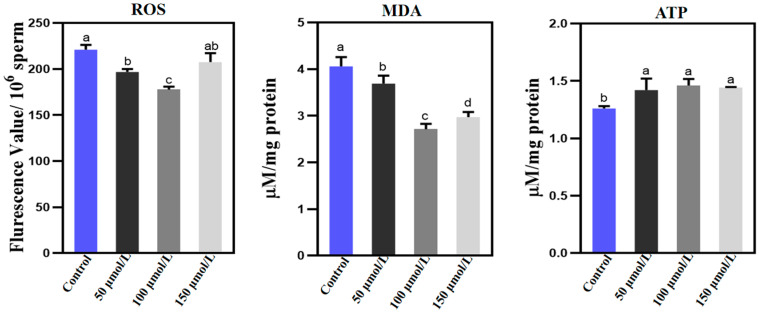
Examination of ROS, MDA, and ATP levels in the sperm of Holstein bulls following treatment with varying concentrations of PQQ during the freezing and thawing processes. Control: no PQQ treatment; PQQ: 50, 100, and 150 µmol/L PQQ/1.0 × 10^8^ sperm. Values are presented as mean ± SD (*n* = 3); columns with different lowercase letters indicate significant differences (*p* < 0.05).

To investigate the underlying mechanisms behind the enhancement of sperm quality induced by PQQ, we conducted a comprehensive evaluation of ROS, MDA, and ATP concentrations. These metrics serves as indicative markers of sperm oxidative stress and metabolic activity during the freeze–thawing process (Figure 3). Compared to the control group, treatment with 100 µmol/L PQQ significantly reduced the ROS levels in frozen–thawed sperm (*p* < 0.05). Additionally, the MDA concentration, a harmful oxidative byproduct, was significantly lower in the PQQ-treated groups compared to the control group (*p* < 0.05), with 100 μmol/L PQQ exhibiting the most pronounced effect. Conversely, all concentrations of PQQ treatment resulted in a significant increase in ATP levels in frozen–thawed sperm (*p* < 0.05).

### 3.4. Effect of PQQ Treatment on Protein Consumption in Frozen–Thawed Holstein Bull Sperm

**Figure 4:**
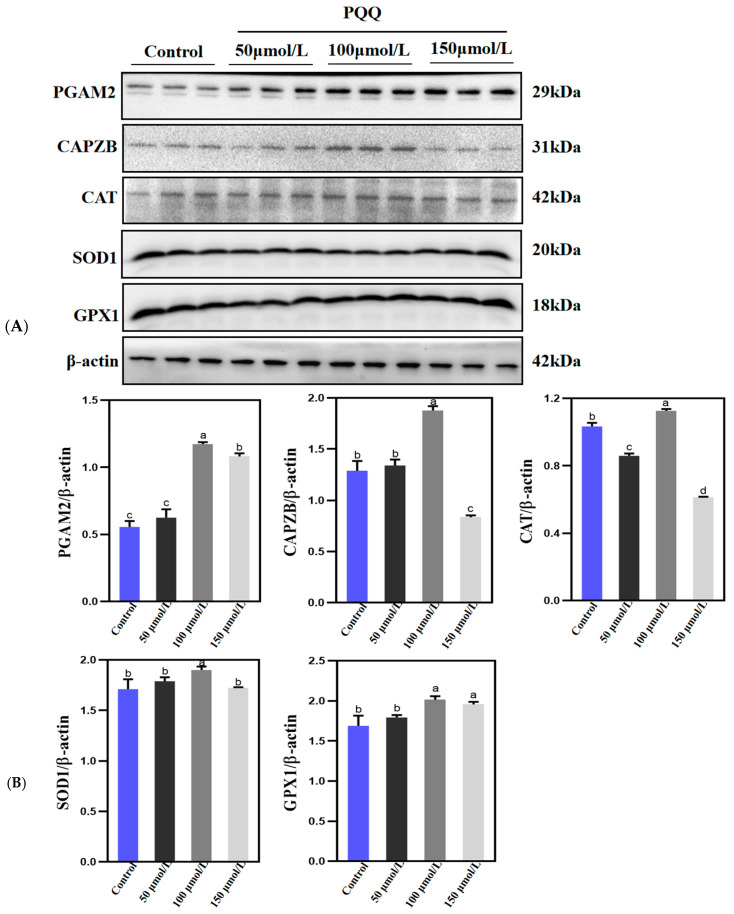
Impact of PQQ treatment on sperm protein consumption during freezing and thawing processes. (**A**) Western blot analysis was conducted to assess the protein levels of key proteins, including PGAM2, CAPZB, CAT, SOD1, and GPX1. (**B**) Grayscale scanning was utilized to quantify the abundance of these proteins (PGAM2, CAPZB, CAT, SOD1, and GPX1). Control: no PQQ treatment; PQQ: 50, 100, 150 µmol/L PQQ/1.0 × 10^8^ sperm. Data are presented as mean ± SD (*n* = 3); columns with different lowercase letters indicate significant differences (*p* < 0.05).

To gain a deeper understanding of how PQQ treatment enhances sperm quality and mitigates oxidative damage, we delved into the investigation of the abundance of proteins that are intricately associated with the structural integrity, metabolic processes, and stress-coping mechanisms within sperm cells. The structural proteins PGAM2 and CAPZB, along with the anti-oxidative stress-related proteins CAT, SOD1, and GPX1, were detected in this study, as illustrated in Figure 4. The results indicated that the protein levels of PGAM2, CAPZB, CAT, SOD1, and GPX1 were significantly elevated in the 100 µmol/L PQQ-treated group compared to the control group, achieving statistical significance (*p* < 0.05). Additionally, the protein levels of PGAM2 and GPX1 were significantly higher in the 150 µmol/L PQQ-treated group than in the control group (*p* < 0.05). However, the protein levels of PGAM2, CAPZB, SOD1 and GPX1 did not show a significant increase in the 50 µmol/L PQQ-treated group compared to the control group (*p* > 0.05).

## 4. Discussion

In the present investigation, varying concentrations of PQQ were utilized as a pretreatment measure for the sperm of Holstein bulls, with the objective of enhancing the effectiveness of cryopreservation. The findings of our study indicated that PQQ significantly improved both the total motility and the structural integrity of the membrane and acrosome in frozen–thawed Holstein bull sperm. Further investigations revealed that PQQ provided protection to sperm from damage during the freezing process by reducing oxidative stress.

The cryopreservation technique for bovine semen has been well established and recognized for over half a century. The utilization of AI with frozen semen is essential in the breeding and selection processes, significantly contributing to the enhancement of cattle production. However, sperm cryopreservation leads to the formation of intracellular ice crystals, osmotic stress, and chilling injury [27], all of which result in reduced membrane fluidity, exacerbated oxidation reactions, and decreased sperm membrane integrity, acrosome integrity [28], and fertilization capacity [15]. Generation and accumulation are expected outcomes during in vitro sperm storage. Notably, the unsaturated fatty acids present in the sperm membrane are particularly susceptible to oxidation by ROS, which compromises the integrity of both the membrane and the acrosome [29,30]. Seminal plasma antioxidants play a crucial role in protecting sperm from oxidative stress [31]. In the current investigation, frozen–thawed sperm from Holstein bulls in the PQQ supplement group exhibited higher values for both membrane integrity and acrosome integrity compared to the control group. Conversely, the control group showed elevated levels of ROS relative to the PQQ treatment group. Therefore, PQQ supplementation can enhance the plasma membrane integrity of bull sperm and acrosome integrity by mitigating the damage caused by ROS during the freezing and thawing processes. As a potent antioxidant, PQQ has consistently been demonstrated to reduce ROS in animal reproduction. Furthermore, it has been reported that the administration of PQQ significantly improved the semen quality in aging layer breeder roosters, as reported by Long et al. in 2022 [22].

Furthermore, PQQ supplementation also reduced MDA and ROS levels in ram sperm while simultaneously increasing ATP levels under 4 °C storage conditions [23]. Similar results were observed in our current study, where PQQ demonstrated a significant and substantial effect in mitigating the decline in ATP levels and the increase in MDA levels noted in bull sperm during freezing. Mitochondria are the primary source of ATP production, which is crucial for sustaining sperm motility [32]. During the freezing and thawing process, semen undergoes substantial biochemical changes, including a reduction in ATP and ADP synthesis [33]. In rats, the addition of ATP to the thawing buffer markedly aids in preserving the functionality of sperm cells that have experienced freezing and thawing [34]. ATP is believed to play a critical role in maintaining the electrochemical balance across the sperm cell membrane, which is essential for preserving both the structural integrity and metabolic activity of the sperm. MDA is the final degradation product resulting from the oxidation of sperm lipids, commonly known as lipid peroxidation (LPO) [35]. Currently, MDA concentration is primarily regarded as an indicative marker of oxidative stress [36]. When aging layer breeder roosters were administered PQQ.Na_2_ supplementation, a significant reduction in MDA concentration in seminal plasma was observed [22]. Therefore, PQQ supplementation has positive effects on enhancing sperm quality and responding to oxidative stress, suggesting potential applications for improving reproductive health and animal reproductive efficiency.

The capping actin protein of muscle Z-line beta subunit (CAPZB) is a pivotal component of the actin cytoskeleton [37] and plays an active role in stabilizing the sperm skeleton network, as well as in the processes of sperm capacitation and the acrosome reaction [38]. The high expression of the CAPZB gene in the testis is likely closely related to its essential functions in spermatogenesis and testicular development [39]. Building on the observed improvement in sperm quality following PQQ treatment, we investigated the abundance of CAPZB to evaluate the extent of structural compromise in frozen–thawed sperm. The findings of this study revealed that PQQ treatment mitigated the loss of CAPZB protein in bull sperm during the cryogenic process. This observation not only highlights the effectiveness of PQQ in preserving the structural integrity of bull sperm during freezing but also provides valuable insights into the protective mechanisms of PQQ on sperm quality. PGAM2, a crucial class of enzymes, plays an indispensable role in the glycolytic pathway. It is not only a key player in this biological metabolic process, but changes in its content directly reflect the current state of the tricarboxylic acid (TCA) cycle, making it an important factor in the study of cellular energy metabolism [40]. In studies involving pigs and gazelles, it has been reported that cholesterol-loaded cyclodextrin (CLC) treatment reduces the negative impact of freezing on sperm glycolysis by decreasing the degradation of PGAM2 [28,41]. We observed that the PGAM2 levels in bull sperm pretreated with PQQ were elevated compared to the control group following the thawing process. This finding indicates that PQQ exerted a protective effect on PGAM2 during the freezing and thawing stages, thereby contributing to the enhanced motility and ATP levels observed in the pretreated bull sperm.

Our study also found that PQQ alleviated the decline in the expression of antioxidant genes, specifically superoxide dismutase 1 (SOD1), glutathione peroxidase 1 (GPX1), and catalase (CAT) in bull sperm following freeze–thawing. These results suggest that the increase in ROS induced by cryopreservation, along with the corresponding decrease in antioxidant gene expression, may be significant factors influencing sperm quality and function. SOD1, GPX1, and CAT play crucial roles in protecting against ROS by effectively neutralizing harmful free radicals, thereby preventing cellular damage [42]. SOD1 is widely regarded as a protective enzyme in male germ cells, playing a vital role in maintaining sperm motility. Its activity has been positively correlated with the capacity of sperm cells to withstand stress during cryopreservation [43]. GPX1 is present in both sperm and seminal plasma, with the highest concentrations found in the mitochondria, where it exhibits protective properties against ROS generated from aerobic metabolism, known to be highly toxic to cells [44,45]. It is well-documented that the incorporation of GPX as an enzymatic antioxidant into a nano lecithin-based extender can significantly enhance both the post-thaw quality and in vitro fertility of bull sperm [46]. Furthermore, CAT supplementation in thawed bull spermatozoa mitigated the effects of oxidants, thereby protecting spermatozoa against ROS and improving both sperm motility and DNA integrity during incubation [47].

During the freezing and thawing process of bull sperm, the application of PQQ treatment significantly reduced the consumption of key proteins, including CAPZB, PGAM2, SOD1, GPX1, and CAT. This reduction ensured the optimal functionality of the sperm upon thawing. Notably, these proteins play a crucial role in the intricate architecture and physiological performance of sperm, encompassing the sperm’s skeletal network, ATP metabolic machinery, and the reduction in ROS.

## 5. Conclusions

The results presented herein demonstrate that the addition of PQQ to bull semen during cryopreservation enhances sperm motility, acrosome integrity, and membrane integrity. PQQ effectively reduced levels of ROS and MDA, while simultaneously increasing the ATP levels in frozen–thawed bull semen. Further analysis of protein levels via Western blotting revealed that PQQ treatment diminished the consumption of PGAM2, CAPZB, CAT, SOD1, and GPX1 proteins in sperm following freeze–thawing. It is therefore recommended that PQQ supplementation be incorporated during the storage of frozen bull sperm, as it is expected to significantly enhance bovine reproductive performance.

## Figures and Tables

**Table 1:** Primary antibodies and secondary antibody utilized in Western blotting.

Parameter	Antibody Name	Host Species	Dilution	Vendor	Code
Primary antibodies	CAT	Rabbit	1:2000	Solarbio	K002366P
	CAPZB	Rabbit	1:2000	Solarbio	K005660P
	PGAM2	Rabbit	1:2000	Solarbio	K005552P
	SOD1	Rabbit	1:5000	proteintech	10269-1-AP
	GPX1	Rabbit	1:5000	cell signaling	#3206
	β-actin	Rabbit	1:2000	Proteintech	81115-1-RR
Secondary antibody	Goat Anti-Rabbit	Goat	1:2000	Proteintech	SA00001-2

## Data Availability

None of the data were deposited in an official repository. All data generated during the study are available from the corresponding author by request.
